# Disruption of 24-Hour Rhythm in Intraocular Pressure Correlates with Retinal Ganglion Cell Loss in Glaucoma

**DOI:** 10.3390/ijms22010359

**Published:** 2020-12-31

**Authors:** Vladimir Neroev, Tatyana Malishevskaya, Dietmar Weinert, Sergei Astakhov, Sergey Kolomeichuk, Germaine Cornelissen, Yana Kabitskaya, Elena Boiko, Irina Nemtsova, Denis Gubin

**Affiliations:** 1 Helmholtz Research Institute of Eye Diseases, 105062 Moscow, Russia; sekr@igb.ru (V.N.); malishevskoff@yandex.ru (T.M.); 2 Institute of Biology/Zoology, Martin Luther University, 06108 Halle-Wittenberg, Germany; dietmar.weinert@zoologie.uni-halle.de; 3 Department of Ophthalmology, Pavlov First Saint Petersburg State Medical University, 197022 St. Petersburg, Russia; astakhov73@mail.ru; 4 Laboratory of Genetics, Institute of Biology of the Karelian Science Center of the Russian Academy of Sciences, 185910 Petrozavodsk, Russia; sergey_kolomeychuk@rambler.ru; 5 Halberg Chronobiology Center, University of Minnesota, Minneapolis, MN 55455, USA; corne001@umn.edu; 6 Сenter for Genomic Technologies, Northern Trans-Ural State Agricultural University, 625003 Tyumen, Russia; yanakabickaya@yandex.ru (Y.K.); egboyko@yandex.ru (E.B.); 7 State Autonomous Health Care Institution Tyumen Regional Ophthalmological Dispensary, 625048 Tyumen, Russia; 74174186@mail.ru; 8 Department of Biology, Medical University, 625023 Tyumen, Russia; 9 Tyumen Cardiology Research Center, Tomsk National Research Medical Center, Russian Academy of Science, 634009 Tomsk, Russia

**Keywords:** glaucoma, intraocular pressure, retinal ganglion cells, optical coherence tomography, circadian, temperature, angiotensin converting enzyme gene

## Abstract

Parameters of 24-h rhythm in intraocular pressure (IOP) were assessed in patients with stable or advanced primary open-angle glaucoma (S-POAG/A-POAG) and referenced to the phase of “marker” circadian temperature rhythm of each patient. Body temperature and IOP were measured over a 72-h span in 115 participants (65 S-POAG and 50 A-POAG). Retinal Ganglion Cell (RGC) damage was assessed by high-definition optical coherence tomography. The 24-h IOP rhythm in A-POAG patients peaked during the night, opposite to the daytime phase position in S-POAG patients (*p* < 0.0001). The 24-h IOP phase correlated with RGC loss (*p* < 0.0001). The internal phase shift between IOP and body temperature gradually increased with POAG progression (*p* < 0.001). Angiotensin converting enzyme Alu-repeat deletion/insertion (ACE I/D) emerged as a candidate gene polymorphism, which may play a role in the alteration of the circadian IOP variability in advanced glaucoma. To conclude, a reliable estimation of the 24-h rhythm in IOP requires the degree of RGC damage to be assessed. In advanced POAG, the 24-h phase of IOP tended to occur during the night and correlated with RGC loss, being progressively delayed relative to the phase of temperature.

## 1. Introduction

An elevated intraocular pressure (IOP) is an important clinical symptom of glaucoma. “Elevated” needs to be qualified, however, since IOP undergoes a circadian (about 24-h) variation. Because IOP is measured using different methods, under different experimental conditions and health status, there is no consensus regarding the circadian characteristics of IOP. In part, ambiguities stem from reported differences in the kind and degree of severity of glaucoma. Multiple exogenous factors, such as posture, physical activity, and diet [1,2,3,4], affect IOP, including whether it is measured at home or in a hospital setting [5]. Not only does the 24-h IOP average, but also its circadian amplitude and phase characteristics depend on age [1,2,6,7], body and head position [6,7], methods of measurement, and different forms of glaucoma [8]. Three approaches currently exist to evaluate IOP: (1) office IOP; (2) self-tonometry; and (3) contact lens sensor monitoring (CLS). The different methods have both pros and cons. IOP measured in the clinic is most rigorously studied in large cohorts but suffers from the inherent unavailability of nighttime data. Measurements outside office hours are possible by self-tonometry, but sampling has remained scarce and limited. Dense sampling, also during sleep, is readily obtained by CLS monitoring. Many studies based on office measurements or self-tonometry showed that, in healthy humans, IOP peaks in the morning or early during the daytime hours [7,8,9,10,11]. Controlled laboratory studies on young healthy volunteers in either a constant supine position [12] or a constant sitting position [13] showed an early morning phase, with propensity to a nocturnal phase in a substantial percentage of individuals. The recently introduced contact-lens-sensor monitors may resolve inconsistencies in the determination of the phase position of the 24-h IOP rhythm in clinical health. With this method, IOP typically follows a sinusoidal pattern peaking during the night [14,15,16,17], but IOP is estimated in millivolts (mV) rather than in mmHg. CLS monitors fluctuations related to IOP in the circumferential curvature of the corneoscleral region via an electric signal sensing-resistive strain [2]. Studies comparing IOP obtained simultaneously by CLS and with a pneumatometer bilaterally in two random eyes showed a similar nocturnal phasing [18]. However, 24-h variations of the two eyes in the same patients measured by these techniques were not correlated, prompting the authors to conclude that the two devices should not be considered to be interchangeable, and that current CLS technology does not yet provide a reliable estimate of 24-h IOP variability [2,18]. To summarize, neither the “ideal technology” for IOP surveillance, nor a perfect algorithm for decision making in interpreting IOP variability is currently established [2]. Additionally, reference standards for IOP parameters have not yet been established.

IOP rhythmicity depends on complex factors that are involved in the maintenance of dynamical balance in aqueous humor. Most of these factors are characterized by 24-h rhythms but are not yet fully explained [1]. Overall, the predominance of a nocturnal or early morning peak in the 24-h IOP variation is thought to be typical for healthy eyes. It is also typical for patients with normal-tension glaucoma [1,2]. However, a daytime peak was often observed in a substantial percentage of participants. Activity and body position influence IOP, also when it is measured by CLS. In the supine position, IOP is increased throughout the 24-h day by almost 5 mmHg in both young and old healthy volunteers [7]; IOP values are more than 20% higher in the supine position in over 35% of patients [19]. As such, different head positions between diurnal (sitting) and nocturnal (supine) measurements might have overestimated nocturnal IOP values and masked IOP’s endogenous circadian variation.

For other forms of glaucoma, 24-h IOP rhythms are even less well understood. In elderly patients, including those with primary open angle glaucoma (POAG), it can be more difficult to determine the typical phase position [20]. In different forms of glaucoma, including POAG, IOP variability tends to be higher and the reproducibility of its dynamic characteristics is lower [21]. In glaucoma, different factors (including older age, higher mean IOP, larger inter-individual differences in mean IOP, and effect of different treatments) account for a greater instability of IOP rhythm parameters, notably the phase of its 24-h rhythm. With advancing age, the phase of the 24-h IOP rhythm tends to shift to later hours, being phase-delayed [7], contrasting with circadian rhythms of most other variables, which are usually phase-advanced [22]. POAG progression is associated with interrelated issues: resistance to IOP-lowering treatment and increasing damage to Retinal Ganglion Cells (RGCs), which may directly impact the 24-h IOP rhythm [23,24]. This situation requires targeted chronobiological studies.

In this study, we investigate IOP variability and examine parameters of its 24-h rhythm in patients with stable and advanced POAG. We also examine the dependence of 24-h IOP characteristics of POAG patients on the degree of their RGC damage. Finally, we evaluate the intrinsic phase angle between IOP and marker circadian rhythms, namely temperature and mean phase of self-reported sleep. This study was intentionally performed under real-life ambulatory conditions in order to look for manifestation of the disease and prevent possible synchronization that may stem from standardized conditions.

## 2. Results

**Figure 1:**
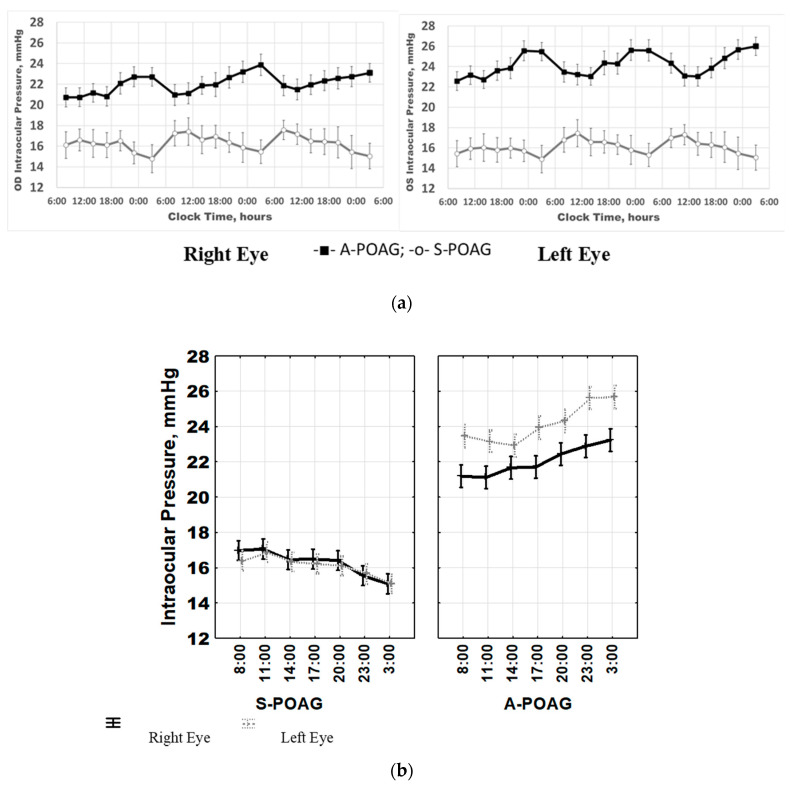
(**a**) Intraocular Pressure (IOP) 72-h patterns in stable primary open-angle glaucoma (S-POAG) and in advanced POAG (A-POAG) for the right eye and the left eye (OD/OS). ANOVA showed a significant time*group interaction for OD (F_(20, 2373)_ = 4.247, *p* < 0.0001) and OS (F_(20, 2373)_ = 4.385, *p* < 0.0001), indicating that the two groups have different time-dependent patterns in both eyes. (**b**) Intraocular pressure (IOP) 24-h patterns for the right and the left eye. MANOVA confirms high similarity of 24-h patterns between both eyes in both groups (F_(12, 4802)_ = 0.588, *p* = 0.740). In A-POAG, mean hourly IOP values were consistently higher in the left eye than in the right eye, *p* < 0.0001.

Overall, the study included 230 eyes from 115 patients; there were 130 eyes from 65 patients diagnosed with S-POAG and 100 eyes from 50 patients diagnosed with A-POAG. The two groups did not differ in mean age, body mass index, or gender; for details, see Table 1. S-POAG and A-POAG groups differed in the extent of damage to the RGCs, assessed by High Definition Optical Coherence Tomography, HD-OCT: GLV was 5.95 ± 1.84% for S-POAG and 24.26 ± 5.09% for A-POAG (*p* < 0.0001). RGC function was proportionally compromised in A-POAG patients, as measured by the decline of PERG P50 A (*Pattern ERG main peak Amplitude*): 2.24 ± 0.85 μV in S- POAG vs. 1.28 ± 0.66 μV in A-POAG (*p* < 0.0001), Table 1. Notably in the A-POAG group, the left eye had a more profound damage and loss of RGC function compared to the right eye (higher RGC GLV % and lower P50 PERG A, *p* < 0.0001 for both indices), Table 1. This difference can be caused, at least in part, by the higher IOP of the A-POAG group (*p* < 0.0001), Table 1, Figure 1a, and the higher IOP of the left eye in A-POAG (*p* < 0.0001), Table 1, Figure 1b. Differences in IOP between the right and left eyes in the A-POAG group were statistically significant throughout the 24-h span, Figure 1b. All POAG patients selected for chronobiological studies received local anti-hypertensive treatment as described in Methods. Results from MANOVA showed no significant effects of treatment on 24-h IOP patterns of POAG patients on different treatment modalities, as shown in Table S1: there were no significant differences in the IOP pattern depending on the type of treatment, either medication or surgery. Patients in the A-POAG group can be regarded as non-responders to treatment, as their IOP did not reach the target values on treatment.

### 2.1. IOP Variability and Circadian Rhythm: Raw vs. Normalized Data Analysis

**Figure 2:**
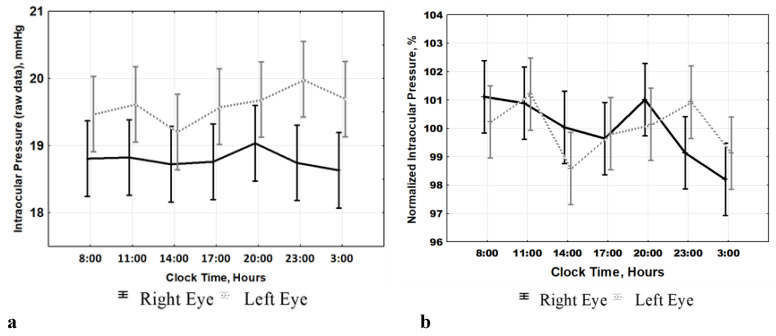
Normalized (**b**) but not raw (**a**) data analysis allows detection of a significant effect of time on intraocular pressure in primary open angle glaucoma pooled cohort. Left: ANOVA does not validate time effect of intraocular pressure (IOP) raw data of the whole primary open angle-glaucoma (POAG) cohort (**a**). Right: Normalized data (percentages of the individual IOP averages (IOP%)) help reduce large inter-individual/inter-group differences in IOP mean values and allow validation of time effect (**b**). Details in text.

Figure 1 and Figure 2 and Table 2 depict results of a comparative analysis of IOP mean values, variability and 24-h rhythm parameters between the two groups. A 24-h IOP rhythm was detected in most S-POAG and A-POAG patients. However, a time effect on raw IOP data from the entire POAG cohort was not detected by ANOVA (right eye: F_(6, 2408)_ = 0.232, *p* = 0.966; left eye: F_(6, 2408)_ = 0.588, *p* = 0.740), Figure 1a. The 24-h IOP patterns for the right and left eyes were highly similar in both groups, Figure 1b, as shown by MANOVA: F_(12, 4802)_ = 0.588, *p* = 0.740. In the A-POAG patients, the mean hourly IOP values were consistently higher in the left eye than in the right eye, *p* < 0.0001, Figure 1b. Two main confounding factors were responsible for the discrepancy between significant individual rhythms and absence of rhythm in the whole POAG cohort. Firstly, there were extensive inter-individual and inter-group differences in mean IOP values. Secondly, there were pronounced time-dependent differences between the S-POAG and A-POAG groups.

**Figure 3:**
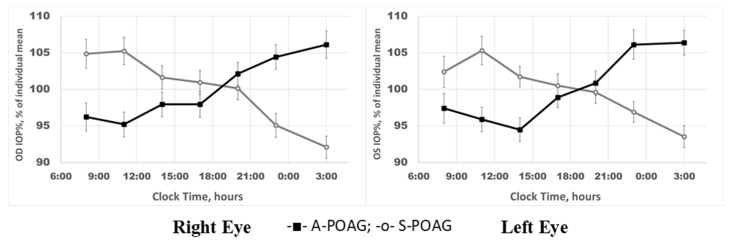
Distinctive 24-h patterns of intraocular pressure (IOP) in stable primary open-angle glaucoma (S-POAG) and in advanced POAG (A-POAG) in both the right eye and left eye (OD/OS). ANOVA showed a significant time*group interaction for OD (F_(6, 2401)_ = 51.52, *p* < 0.0001) and OS (F_(6, 2401)_ = 45.99, *p* < 0.0001), indicating that the two groups have time-dependent patterns showing similar differences in both eyes. Note: Normalized data (percentages of individual IOP averages, IOP%) were used to reduce large inter-individual variability.

The first confounder can be eliminated by normalizing the raw data by expressing them as percentages of individual IOP mean values as described in Methods. A significant time effect was indeed found by ANOVA for the entire POAG cohort once the normalized data are analyzed (right eye: F_(6, 2408)_ = 2.889, *p* = 0.008; left eye: F_(6, 2408)_ = 2.072, *p* = 0.053), confirming that IOP varies along the 24-h scale, Figure 2b. The second factor became obvious after running a two-way ANOVA: the time*group interaction provides evidence that the time patterns of the S-POAG and A-POAG groups were distinctively different. This significant time*group interaction was found when analyzing the data over the 72-h span (right eye: F_(20, 2373)_ = 4.247, *p* < 0.0001; left eye: F_(20, 2373)_ = 4.385, *p* < 0.0001), Figure 2a, or after stacking the data over a 24-h span (right eye: F_(6, 2401)_ = 51.52, *p* < 0.0001; left eye: F_(6, 2401)_ = 45.99, *p* < 0.0001), Figure 3.

### 2.2. IOP Circadian Phase in POAG Progression and Its Relationship to Marker Circadian Rhythm (Body Temperature, Tb) and Sleep

**Figure 4:**
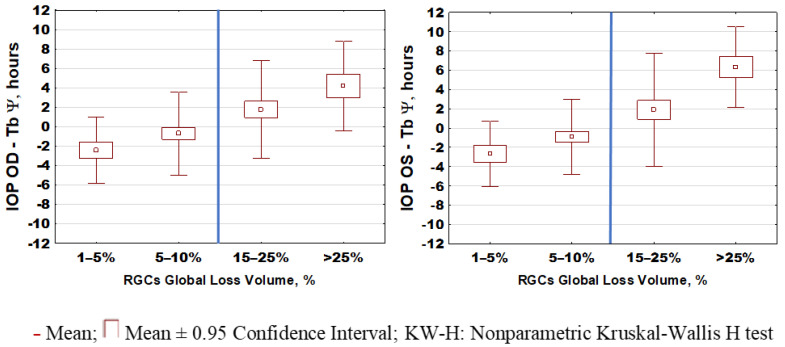
Gradual misalignment between intraocular pressure (IOP) and circadian marker rhythm (temperature, Tb) associates with gradual loss of retinal ganglion cells (RGCs) in primary open-angle glaucoma progression. Intrinsic phase angle, ψ, between IOP and Tb is gradually shifted from IOP preceding Tb to IOP lagging Tb as retinal ganglion cell loss gradually progresses in primary open-angle glaucoma; right eye (OD): KW-H_(3;115)_ = 18.33, *p* = 0.0004; left eye (OS): KW-H_(3;115)_ = 33.47, *p* < 0.0001. Blue vertical lines demark the threshold between S-POAG and A-POAG groups.

**Figure 5:**
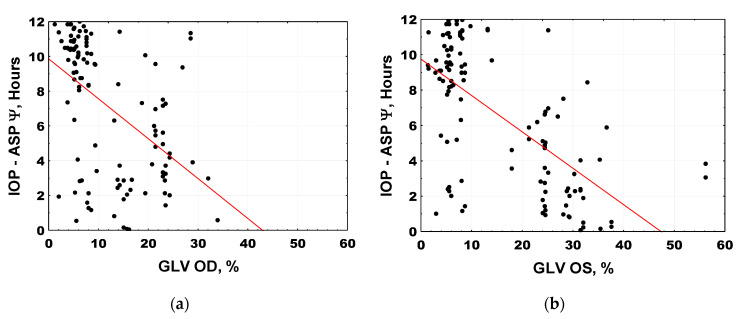
Progressive IOP-to-ASP circadian phase shift (IOP phase propensity to sleep mid-phase) is associated with higher retinal ganglion cell global loss volume (GLV); right eye (OD): r = −0.489, *p* < 0.0001; left eye (OS): r = −0.653, *p* < 0.0001. ψ—intrinsic phase angle between respective eye IOP and ASP; ASP—average sleep phase. Global Loss Volume (GLV%) of the right eye (OD)(**a**), Global Loss Volume (GLV%) of the left eye (OS) (**b**).

Different time patterns of IOP in the two groups of patients were further explored for phase characteristics, and for phase agreement with Tb’s marker circadian phase and self-reported sleep phase. Not only were IOP phase relations with Tb and sleep different between S-POAG and A-POAG patients, phase changes were also related to RGC GLV within these groups. Figure 4 illustrates the changes in intrinsic phase angle between IOP and Tb (“marker” circadian rhythm). A gradual internal 24-h phase misalignment between IOP and Tb occurred in POAG and further increased with its progression. Earlier IOP-to-Tb phase in mild-stage glaucoma (individuals with mean GLV% < 5%) is followed by a neutral phase position in S-POAG, with GLV% of 5–10%, and by a progressively later IOP-to-Tb phase position in A-POAG. The deviation of the circadian phase angle, Ψ (of IOP from Tb), correlated highly significantly with RGC global loss (GLV%) in the entire POAG cohort for each eye (right eye: r = 0.312; *p* = 0.0007; left eye: r = 0.482; *p* < 0.0001). The intrinsic misalignment between IOP and Tb occurred even though the circadian phase of Tb is itself delayed with POAG advancement [25]. Furthermore, this delay was greater than that of the average sleep mid-phase (ASP), as previously described in detail elsewhere [25]. An even more pronounced IOP phase shift was found in relation to the self-reported ASP (right eye: r = −0.489; *p* < 0.0001; left eye: r = −0.653; *p* < 0.0001), Figure 5, Table 2. Figure 5 illustrates the shift in the 24-h IOP rhythm in relation to ASP based on individual data; the distribution of relative phases is shown in relation to individual GLV values. The IOP phase of the A-POAG group was shifted towards mid-sleep by 4 h 22 min for the right eye and by 5 h 29 min for the left eye, Table 2. In the A-POAG group, the IOP circadian phase propensity to ASP for the left eye was greater than for the right eye (*p* = 0.05), Table 2, as was GLV% of the left eye (*p* < 0.0001), Table 1.

The mean phase of the 24-h IOP rhythm of the A-POAG group was inversed to a nocturnal position, contrasting with a diurnal position of the S-POAG group (*p* < 0.0001). In the whole POAG cohort, the lag between the circadian phase of IOP and ASP is significantly correlated with GLV and the mean IOP of the respective eye. The association with RGC damage was stronger than with the mean IOP, as confirmed by a backward stepwise multiple regression model, showing that propensity of the IOP phase towards ASP correlated predominantly with the RGC GLV% (right eye: r = 0.65, *p* < 0.0001; left eye: r = 0.49, *p* < 0.0001).

### 2.3. Candidate Gene Polymorphisms Involved in IOP 24-h Variability Modulation

**Figure 6:**
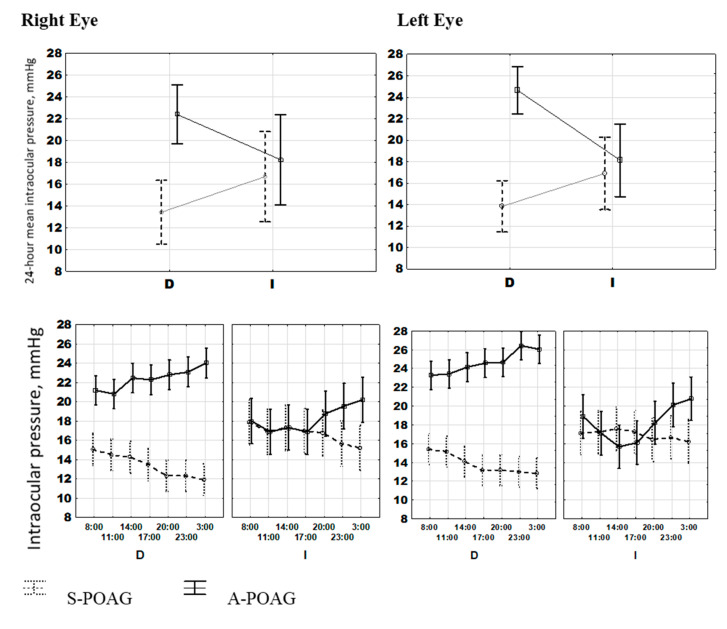
Angiotensin converting enzyme (ACE) gene I/D polymorphism—a putative cofactor predisposing to higher intraocular pressure (IOP) in advanced POAG that may influence architecture of IOP 24-h variability. ACE Alu-repeat deletion (D-allele) associates with higher IOP in advanced primary open-angle glaucoma. ANOVA revealed significant allele*group interaction: right eye: F(1, 15) = 4.99, *p* = 0.041; left eye: F(1, 15) = 12.55, *p* = 0.003. S-POAG—stable primary open-angle glaucoma; A-POAG—advanced primary open-angle glaucoma.

Among eight investigated genes, angiotensin converting enzyme, ACE (I/D: Alu-repeat deletion/insertion), emerged as a candidate polymorphism, which may play a role in the modified IOP 24-h variability. ACE intron-localized Alu-deletion, D-allele may be linked to the elevated mean IOP in advanced POAG and determine its specific 24-h pattern. The ANOVA revealed a significant allele*group interaction (right eye: F_(1, 15)_ = 4.99, *p* = 0.041; left eye: F_(1, 15)_ = 12.55, *p* = 0.003) (see Figure 6). Alternatively, this polymorphism can indicate resistance to IOP-lowering treatment. A difference in 24-h IOP patterns between D-carriers (DD and ID genotypes combined) and non-carriers (II genotype) in the A-POAG group is not likely caused by a difference in GLV. Indeed, GLV was similar between D-carriers and non-carriers in the S-POAG and A-POAG groups (see Figure S1). ACE Alu-repeat insertion-deletion (I/D) polymorphism may also specifically impact 24-h IOP patterns, depending on POAG progression. MANOVA showed a significant effect for time*group*allele interaction for the left eye (F_(6, 371)_ = 3.27, *p* = 0.004) (see Figure S2). Characteristics of participants in the S-POAG and A-POAG groups engaged in the genetic polymorphism trial study were generally similar to the whole cohort (see Table S2).

## 3. Discussion

This study led to several important findings. Firstly, IOP data from POAG cohorts must be normalized when group analyses are performed; otherwise, high inter-individual variability may prevent the detection of a circadian rhythm in IOP. Secondly, our results point to specific alterations in the circadian rhythm of IOP, which occur gradually from mild to advanced POAG. They are predominantly associated with the progressive damage and dysfunction in RGC. A greater RGC damage in the left eye of A-POAG patients was linked to a greater disruption of the circadian IOP phase in this eye. Lastly, a polymorphic variant of the *ACE* gene, 16th intron 289-nucleotides *Alu* repeat deletion, D-allele emerged as a candidate polymorphism, which may play a role in the alteration of the 24-h variability in IOP, and cause resistance to IOP-lowering treatment.

Increasing RGC damage and dysfunction seen with POAG development and progression may account for the reduced amplitude of light signaling, i.e., the reduced capacity of the melanopsin-containing, intrinsically photosensitive RGCs (ipRGCs) to perceive and transduce light signals to the SCN (central clock). A vicious circle may follow, also including a reduced self-chosen daytime activity, natural light exposure, and compromised sleep quality. In POAG and its progression, RGCs are damaged [23,26,27,28,29]; the process involves ipRGCs as well, though their damage occurs at more advanced disease stages [28,29]. Since ipRGCs are essential for non-visual signal transduction to the hypothalamic SCN, the central oscillator of the biological clock [30], the reception and transduction of light signaling are compromised. We show herein that the number of RGCs decreases with progressing POAG. A recent study demonstrated histologically that this particularly concerns melanopsin-expressing RGCs. In mild-staged glaucoma, the ipRGC density is comparable to that of age-matched controls, whereas at severe stages, a 3-fold loss was observed [28].

Conditions of reduced quality of the transmission of entraining impulses to the SCN are prerequisites for altered light-driven synchronization of the circadian rhythms [23,24]. Damage to the ipRGCs is one of the factors predisposing to chronic circadian disruption and misalignment between the intrinsic biological clock and a principal external time cue such as light [23,24,29]. Internal phase misalignment has various adverse effects on physical and mental well-being and sleep parameters.

Variability in IOP and its 24-h pattern in health and different forms of glaucoma remain a matter of debate [2] since their nature and mechanisms are not fully understood. The phase of the circadian IOP rhythm and its relative phase angle to a “marker” circadian rhythm, such as temperature, vary in mammals, depending on the ambient lighting. Human studies in healthy young adults found that the phase characteristics of the IOP rhythm vary significantly. Authors who carried out research on circadian IOP rhythms in diurnally active animal models obtained different results on their phasing. For example, a diurnal circadian IOP phase was observed in a horse model [31,32], while in a rabbit, the circadian IOP rhythm had a definite nocturnal phasing [33]. Authors also came to different conclusions concerning the nature of the circadian IOP rhythm. Bertolucci et al. [31] and Piccione et al. [32] came to the conclusion that the circadian IOP rhythm is driven by the central oscillator, while another group found in a mouse model that it free-runs with a period of unequal length [34]. These results prompt the proposition that the circadian IOP rhythm may be entrained by an oscillator, which is different from the suprachiasmatic nuclei of the hypothalamus. It was suggested that the circadian IOP oscillator may be located within the organ of vision [35]. However, further studies in a mouse model aimed at assessing whether, like the retina, the mammalian ciliary body and IOP clocks have an intrinsic ability to be synchronized by the light/dark cycle. These results prompted the authors to a drew-back conclusion that the IOP rhythm is not synchronized locally within the eye, but rather relies on synchronizing signals from the suprachiasmatic nucleus or other sites [36]. This scenario suggests a possible dependence of the circadian IOP rhythm on several synchronizing factors of both exogenous and endogenous origin, with substantial individual differences. Our results assume that in POAG patients the mechanisms of synchronization are deteriorated. Consequently, a phase shift may occur in accordance with the local free-running period, a possibility that was suggested earlier [34].

Circadian IOP patterns differed significantly between patients with different forms of glaucoma and healthy individuals. According to both tonometry and CLS monitoring, glaucoma patients have increased IOP variability [15,21]. Less predictable IOP variability in glaucoma leads to a wider distribution of phases of the 24-h rhythm, both among individuals and on different days in the same individuals. This phenomenon is general for chronobiology of both aging and disease and often corresponds to so-called extra-circadian dissemination [37]. Increased variability is typically linked to intrinsic circadian phase misalignment among circadian rhythms.

Herein, intrinsic misalignment between IOP and Tb occurred even though the circadian phase of Tb was itself delayed as a function of POAG advancement [25]. Whether the nocturnal phase of the 24-h IOP rhythm in advanced glaucoma is a consequence of the disease, or its precursor should be clarified in further studies. The predominance of a nocturnal phase in A-POAG may underline that this type of IOP rhythm is an indication of glaucoma progression. Our results assume that a GLV of RGCs above 10–15% may represent a threshold for the manifestation of an altered phase of the circadian rhythms (IOP and Tb in particular). Results herein also suggest that the ambiguity regarding the phase of the 24-h IOP rhythm may stem from including in different proportions patients at different stages of POAG.

Improved methods of assessing IOP variability should help ensure that this question is effectively answered. In recent years, methods for outpatient observation of the 24-h dynamics in IOP continue to gain popularity [11]. Our results suggest that technical solutions in this area are needed to ensure that measuring IOP at night and during sleep is not burdensome for the patient and not biased from differences in body / head position and other conditions. Such data are of primary interest both for the interpretation of results and for assessing risk factors in the diagnosis of glaucoma progression.

We consistently observed higher IOP values of the left eye in the A-POAG group. Possible causes of higher IOP of the left eye in the A-POAG group are not known. One possibility is that it could derive from stochastic factors, such as the weakened strength of association between the 24-h rhythms and variability of two eyes in advanced glaucoma stages [38]. This situation can result in a significant difference in IOP between the two eyes, as reported for a similarly large cohort, though with higher values of the right eye [39]. Some authors reported that some patients found self-measurements of IOP of the left eye with the iCare device more difficult [11], possibly influencing the measurements. On the other hand, in the present study, we found no difference in the 24-h patterns of IOP between the two eyes within the A-POAG group for the raw data by ANOVA (F_(6, 2086)_ = 0.908, *p* = 0.488).

Our results suggest that the ACE *Alu* repeat I/D polymorphism may affect the 24-mean value of IOP, the individual resistance to IOP-lowering treatment, and the 24-h IOP variation in POAG patients, Figure S1. Several studies showed that angiotensin and ACE are present in RGCs and the ciliary body, and that they play a role in aqueous humor secretion and outflow [40]. Aqueous humor balance may be the key for understanding rules of circadian IOP rhythmicity [1]. The *Alu* element is one of the main factors causing instabilities in the human genome that are associated with numerous diseases. Intronic modifications of *Alu* repeats in the human genome (deletions or insertions) may affect alternative splicing processes, thereby modifying either the expression rate or protein structure and function [41]. Specifically, the ACE *Alu* repeat deletion vs. insertion (D-allele) is linked to the risk of higher blood pressure [42] that can be explained by different rates of ACE expression [43]. Whether this ACE gene polymorphism may also affect the timing of ACE expression within the eye remains to be elucidated.

There were limitations to this study. IOP data were obtained by self-measurements in a home setting. Hence, body position could have influenced individual patients’ compliance with physician’s instructions. Repeated sampling on relatively large numbers of patients may have mitigated such impact. The single nocturnal IOP measurement may have slightly disturbed its 24-h pattern, but even more obtrusive hourly awakenings reportedly had only subtle to no effect on IOP and sleep parameters [44]. Measurements in a home setting may also have differed from those obtained under standardized laboratory conditions. On the other hand, laboratory conditions could bring an artificial bias to the habitual lifestyle, which in turn could be a consequence of circadian mechanisms linked to the disease itself or to its progression. Results from a home setting may hence constitute a valid approach for clinical applications of the results.

Another point is that ipRGC loss was not specifically analyzed in our study. A previous study confirmed that ipRGC loss is indeed evident in glaucoma, affecting sleep quality [27]. Another histological study demonstrated that ipRGC loss likely occurs in advanced rather than in mild glaucoma [28]. Furthermore, since the ensemble of cells within the retinal system clearly displays inter-dependency [45], damage and dysfunction of not just ipRGCs may have certain consequences regarding circadian alignment. Herein, there were unfortunately no participants with a mean GLV between 10% and 15%. Including this group in future studies could be interesting to investigate the phase behavior of IOP since the putative threshold for abrupt changes is situated within this range.

Any influence of external factors on IOP (physical activity and sleep, ambient light, food and water consumption, etc.) was left largely unexplored. To our knowledge, there have been no controlled studies assessing the endogenous circadian rhythm in IOP performed in constant routine or under forced desynchrony protocols. Studies using different approaches by taking, preferably simultaneously, IOP measurements conventionally (in mmHg) and by contact lens sensor devices (in mV) are warranted to obtain reference standards for circadian rhythm parameters of IOP. Not a single study was found in the literature that considered a marker circadian phase (such as temperature, chronotype, dim light melatonin onset, or actimetry) when assessing the circadian phase of IOP. Our study provides the first insight into such a relationship between IOP and temperature rhythms (see also [25]).

Complex statistical procedures available to deal with data collected from paired organs were not used herein. Instead, our aim was to examine whether there was a simple way of relying on a single eye or on the average value from both eyes that may have been more advantageous than another. Although the different approaches used herein led to similar overall conclusions, further investigations are needed to answer this question.

Our preliminary results on candidate gene polymorphism (ACE I/D) are based on a modest sampling that precludes drawing any definite conclusions yet. Hence, these results are kept under supplementary materials. A logic mechanistic explanation for such a relationship, however, can be offered. Further gene polymorphism studies are needed that also consider interactions with other putative candidate polymorphisms. Additionally, patients diagnosed with hypertension and receiving appropriate treatment could not be excluded, since they constitute the majority of patients with glaucoma. Systemic and local treatment modalities are known to influence IOP 24-h patterns [46,47,48] and affect melatonin production, i.e., beta-blockers [49]. In the present study, though, no significant differences in 24-h IOP patterns of POAG patients with different treatment modalities were found, Table S1.

## 4. Materials and Methods

This cross-sectional study adhered to the tenets of the Declaration of Helsinki and was approved by the Institutional Review Board at the Tyumen Scientific Center of the Siberian branch of the Russian Academy of Sciences (Protocol No. 5, 15 May 2013). The work was included in the research plan of the Federal State Budgetary Institution of Science at the Tyumen Scientific Center of the Siberian branch of the Russian Academy of Sciences (registration number AAAA-A17-117120500038-2). Written informed consent was obtained from all participants according to the order of the Ministry of Health of the Russian Federation, No. 266 (19.03.2003). All patients were examined and diagnosed under the supervision of the State Autonomous Health Institution of the Tyumen Region “Regional Ophthalmological Dispensary” during 2013–2016.

### 4.1. RGC Function and Damage Assessment

Standard automated perimetry (SAP) was performed to assess visual field (VF) with the Humphrey Field Analyzer (Carl Zeiss, Jena, Germany) by using the 30-2 SITA-Standard strategy. The following parameters were obtained: the total photosensitivity of the central VF, mean deviation (MD) and pattern standard deviation (PSD).

Damage to the Retinal Ganglion Cell Complex (RGCC) was measured by means of high-definition optical coherence tomography (HD-OCT) (RTVue-100, Optovue, 2800 Bayview Dr, Fremont, CA, USA). The average amount of GCC loss over the entire GCC map (Global Loss Volume, GLV, %) and the average amount of localized thinning over the entire GCC map (Focal Loss Volume, FLV, %) were estimated. Optic nerve head (ONH) and retinal nerve fiber layer (RNFL) scanning protocols 3.45 were used, *GCC for the RTVue-100* tomograph.

Functional ability of RGC was also assessed by using amplitude of pattern electroretinogram (PERG), a valid method to predict the development and progression of glaucoma [50,51]. In our study, PERG was assessed at three different times of the 24-h cycle, once per day on 3 consecutive days, at 8:00 on the first day, at 14:00 on the second day, and at 20:00 on the third day. No nocturnal data were obtained in view of the need to conduct research in the clinic and of the complexity and burden of the procedure for the patient. Herein, mean values from each patient’s three consecutive measurements were used. PERG was obtained by “Tomey EP 1000” (*Tomey, Japan-Germany*) electroretinography. Electrode-cups were fixed on the lower eyelid. Results were assessed and evaluated based on the International Society for Clinical Electrophysiology of Vision (ISCEV) recommendations [52].

### 4.2. POAG Diagnosis and Progression Criteria

The criteria for selecting patients with POAG were visual acuity 0.5–1.0 (without correction or with correction requiring no more than ±3.0 diopters, and no more than 1 diopter for astigmatism), a transparent lens and no pathology of the macular region of the retina. Criteria for progression of POAG were based on SAP-derived mean deviation (mD) [53] and the dynamic index of the GLV%, according to results from the Optical Coherence Tomography (OCT) [54]. The dynamics of visual functions were assumed to have stabilized in patients with a change in mD by no more than 0.5 decibels (dB) per year, and a decrease in GLV by no more than 2% per year. These patients were assigned to the stable group, S-POAG. In other cases, the process was considered progressive, and patients were assigned to the advanced group, A-POAG. Depending on the dynamics of glaucoma progression criteria, mD (dB) and GLV (%) in early 2014 and late 2016, all patients were divided into two groups: S-POAG (*n* = 289) and A-POAG (*n* = 427). Data from the worst eye were considered in cases of discrepancy.

For further chronobiological studies, 115 (65 S-POAG and 50 A-POAG) patients, matched by gender, age and treatment modalities (Table S1), were selected. All POAG patients selected for chronobiological studies received local anti-hypertensive treatment (prostaglandin F2α analogues, β-blockers, carbonic anhydrase inhibitors) or underwent previous surgery with non-penetrating deep sclerectomy or sinustrabeculectomy. The exclusion criteria were the following: primary open-angle glaucoma of the end stage; other types of glaucoma; severe scarring of the cornea; severe cataract; hereditary eye diseases, inflammatory eye diseases; high-degree myopia; occlusion of the central artery/central retinal vein; and age-related central retinal degeneration. Excluded were also patients with acute coronary or cerebral blood flow disorders; heart rhythm disorders; cancer; mental diseases, including alcohol use disorder; neurodegenerative diseases, such as Alzheimer’s, Parkinson’s, and multiple sclerosis; diabetes mellitus; thyroid disease; shift workers; and patients crossing time zones at least once a month.

Restrictions to daily routine were not advised because they could artificially modify the natural daily routine characteristic of a group. Instructions about the procedures of self-measurements were given to all patients, who were asked to keep a diary reflecting self-reported health, physical activity, food intake, medication, bedtime and time of awakening.

### 4.3. Intraocular Pressure Measurements

IOP was measured 7 times a day (at 8:00, 11:00, 14:00, 17:00, 19:00, 23:00 and 3:00 h) for three consecutive days (72 h), in accordance with Tyumen’s Protocol. This protocol was previously used in numerous chronobiologic studies [25,55,56,57,58] and showed good agreement of 24-h phase and amplitude estimates as compared to more dense sampling protocols and monitoring techniques. It was designed to minimize the impact of repeated measurements on sleep while remaining compatible with obtaining a reliable estimate of amplitude and phase of the 24-h rhythm. Self-measurements of IOP (*n* = 21) in patients were always performed in a vertical (upright) position in order to avoid bias from changing head position, which reportedly influenced resting IOP [19]. Changing body position may also affect the estimation of the 24-h IOP amplitude and phase. Measurements were taken with a portable intraocular pressure tonometer for individual use (ICare ONE, TA02, Icare Finland Oy, Vantaa, Finland). Preliminary training of all patients in the measurement techniques was performed at the glaucoma department of the dispensary. Measurements in the evening and during the night (03:00) were asked to be taken while avoiding ambient light in the room.

Since the average IOP value differed among patients, the data of individual patients were expressed as a percentage of their respective 72-h average in order to normalize the 24-h variation around a mean value of 100%. The normalization algorithm included two steps: first, the mean IOP value over the 72-h time series was calculated for every patient; and second, the IOP values at each time point of the 72-h time series were divided by the mean value obtained in the first step and expressed as a percentage for every individual patient. Inter-individual differences in mean IOP values between the two groups were thus eliminated. Further analysis of the original IOP data in relation to the degree of RGC damage identified distinctive circadian patterns of IOP variability within each POAG cohort.

### 4.4. Body Tempeature (Tb) Measurements

Axillary body temperature (Tb) was measured 7 times per day (at 8:00, 11:00, 14:00, 17:00, 19:00, 23:00 and 3:00 h) on three successive days (72 h) according to Tyumen’s protocol that was previously applied in several studies [25,55,56,57,58]. Tb was measured by mercury thermometer (Amrus AMTD, Amrus Enterprises Ltd., 08863, New Jersey, USA). Measurements to be taken during sleep were requested to be taken by a family member without turning on the external lighting in the room in order to avoid sleep interruption. Circadian rhythm disruption of temperature associated with POAG progression was previously described by us in detail elsewhere [25].

### 4.5. Sleep Assessment

Though assessment of sleep parameters was beyond the main scope of the present article, we decided to add objective data on individual sleep habits. Therefore, personal sleep diaries provided information on basic sleep parameters, e.g., the time of going to bed and time of awakening. Sleep duration was calculated from that data; the mid-time of this span was used as mid-sleep phase. Collected information from three consecutive days was averaged to obtain a mean Actual Sleep Duration (ASD) and a mean Actual Sleep Phase (ASP). Our findings on specific sleep alterations found in association with POAG were recently reported elsewhere [25].

### 4.6. Chronotype Assessment

The chronotype score (CS), which reflects individual habitual preferences in the 24-h activity schedule, was assessed by the Horne–Ostberg Morningness–Eveningness Questionnaire (MEQ) [59].

### 4.7. Genotyping

Always the same operator, who was not aware of the participant’s clinical characteristics, performed the genotyping. Saliva samples were collected via standard protocols from 19 patients, 9 representing the S-POAG group and 10 representing the A-POAG group. Comparative characteristics of patients engaged in the gene polymorphisms study are provided in Table S2. Participants were asked to rinse their mouth with water and wait for ten minutes before saliva collection. Next, they spit into the collection tubes until the saliva level reached the 1 mL line. Handling solution was then added, and the tube was gently shaken by hand for at least ten seconds. DNA was isolated from patients’ samples by DiaGene DNA Isolation Kit (Dia-M; Moscow, Russia) according to the manufacturer’s instructions. The real-time polymerase chain reaction was performed using iCycler Real Time System with iQ5 Manager software of Bio-Rad Laboratories Inc. (Hercules, CA, USA). Polymorphic gene variants were identified by SNP Screen Kit (Syntol; Moscow, Russia) for 8 genes (clock genes: PER2 rs6431590, PER3 VNTR, CLOCK rs 1801260 3111T/C, cryptochrome CRY1 rs12820777; melatonin receptor genes MTNR1A rs34532313, MTNR1B rs10830963, G-protein GNB rs5443; and angiotensin converting enzyme, ACE rs1799752 insertion/deletion). In each reaction, two allele-specific hybridizations were used to detect two alleles of the studied polymorphism, independently on two fluorescence channels (ROX and FAM).

### 4.8. Data Analysis

Individual 24-h rhythms of IOP and Tb were assessed by single cosinor analysis [60]. Each 72-h time series was fitted with a 24-h cosine curve by least squares to yield estimates of the MESOR (M, Midline Estimating Statistics of Rhythm, a rhythm-adjusted mean), 24-h amplitude (A) and acrophase (Ψ, phase angle of the peak of the cosine curve fitted to the data in reference to local midnight). Results were summarized by population-mean cosinor [60]. Rhythm parameters were compared using Bingham’s parameter tests [61]. The data were also analyzed by one-way analyses of variance, ANOVA, and Multivariate analysis of variance, MANOVA, testing for group and/or time effects. Correlation and regression analyses further examined relations between rhythm parameters and indices of RGC damage.

Circadian parameters were correlated with RGC measures from the left and right by different approaches: based on each eye estimate separately, based on the better eye estimates, and based on the two-eyes mean estimates to find out an approach that provides the strongest correlation with parameters of the circadian rhythm. For each individual patient, intrinsic phase angles between IOP (of each eye) and Tb (body temperature circadian marker rhythm) (Ψ = ϕ_IOP_−ϕ_Tb_) (Ψ = ϕ_IOP_−ϕ_Tb_), and between IOP and mean Average Sleep Phase (ASP) (Ψ = ϕ_IOP_−ϕ_ASP_) were calculated, considering that they could not exceed the 24-h half-cycle (12 h). Both raw and normalized IOP data were considered.

STATISTICA 12, SPSS 23.0 and Excel packages were used to run ANOVA (One-way analyses of variance), MANOVA (Multivariate analysis of variance) and tests for significant differences. Normal distribution was checked by Shapiro–Wilk’s W-test. In cases of normally distributed variables (W-test’s *p*-value > 0.05), a 1-way ANOVA was used, with Tukey’s post hoc correction for multiple testing. Otherwise, the Kruskal–Wallis and the Mann–Whitney post hoc tests were used. The level of statistical significance was set at 5%. Exact *p*-values are listed in the text and tables.

## 5. Conclusions

Our results indicate that IOP data must be normalized for population analyses due to the high inter-individual variability in IOP in POAG and more so with its progression. IOP variability becomes more stochastic, with increasingly scattered phases of the 24-h rhythm. IOP phases gradually misalign in relation to the circadian temperature rhythm, and are delayed to night hours in association with progressive loss of RGCs. Hence, the ambiguity of existing results on the 24-h IOP phase may depend on the ratio between the number of patients with different degrees of RGC damage and dysfunction, and on gene polymorphisms.

## Figures and Tables

**Table 1:** General and Clinical Characteristics of Patients with Stable and Advanced Primary Open-Angle Glaucoma (S-POAG and A-POAG).

	Stable POAG	Advanced POAG	*p*-Value
**General Characteristics**			
Age, years.	67.61 (60.05; 75.17)	69.98 (61.83; 78.13)	0.100 ^§^
Number, Sex	*n* = 65, 46 women	*n* = 50, 31 women	0.322 ^#^
BMI, kg/m^2^	24.66 (23.91; 25.41)	24.77 (23.95; 25.60)	0.852 ^§^
**Clinical Characteristics**			
Global Loss Volume mean, %	5.95 (5.50; 6.41)	24.26 (22.82; 25.71)	<0.001 ^§^
Global Loss Volume right eye, %	5.75 (5.32; 6.18)	20.05 (18.43; 21.67)	<0.001 ^§,^*
Global Loss Volume left eye, %	6.15 (5.60; 6.72)	28.49 (26.33; 30.64)	<0.001 ^§,^*
Focal Loss Volume mean, %	3.20 (2.72; 3.68)	10.81 (10.13; 11.48)	<0.001 ^§^
Focal Loss Volume right eye, %	2.95 (2.49; 3.41)	9.30 (8.75; 9.85)	<0.001 ^§,^*
Focal Loss Volume left eye, %	3.31 (2.79; 3.84)	12.31 (11.29; 13.34)	<0.001 ^§,^*
SAP mD mean, dB	−3.42 (−3.92; −2.91)	−11.95(−13.23;−10.67)	<0.001 ^§^
SAP mD right eye, dB	−3.43 (−3.94; −2.92)	−8.04 (−9.71; −6.37)	<0.001 ^§,^*
SAP mD left eye, dB	−3.41 (−4.09; −2.72)	−15.86 (−17.68;−14.03)	<0.001 ^§,^*
PERG P50A mean, μV	2.22 (2.02; 2.43)	1.06 (0.92; 1.21)	<0.001 ^§^
PERG P50A right eye, μV	2.24 (2.03; 2.45)	1.28 (1.09; 1.47)	<0.001 ^§,^*
PERG P50A left eye, μV	2.21 (2.01; 2.41)	0.85 (0.68; 1.02)	<0.001 ^§,^*
IOP right eye mean, mmHg	16.29 (15.56; 17.03)	22.03 (21.00; 23.06)	<0.001 ^§,^*
IOP left eye mean, mmHg	16.10 (15.25; 16.96)	24.16 (23.16; 25.16)	<0.001 ^§,^*

BMI—body mass index; IOP—24-h mean intraocular pressure; SAP mD—Standard Automated Perimetry mean Deviation; PERG P50 A—Pattern Electroretinogram main peak (P50) Amplitude; Tb phi–circadian mean phase of body temperature; Mean (95% confidence range). ^§^ Mann–Whitney U test; ^#^ Pearson’s chi-square test; * statistically significant difference between right and left eyes for A-POAG group.

**Table 2:** Normalized Intraocular Pressure Variability, Circadian and Sleep Parameters of Patients with Stable and Advanced Primary Open-Angle Glaucoma.

	Stable POAG	Advanced POAG	*p*-Value
**IOP Variability and 24-h Characteristics**			
IOP_OD_ SD, %	11.80 (10.58; 13.03)	11.44 (10.07; 12.81)	0.666 ^§^
IOP_OS_ SD, %	11.96 (10.97; 12.96)	10.73 (9.27; 12.20)	0.012 ^§^
IOP_OD_ 24-h Amplitude, %	8.52 (7.21; 9.84)	7.70 (5.93; 9.48)	0.099 ^§^
IOP_OS_ 24-h Amplitude, %	7.33 (6.11; 8.56)	7.62 (5.98; 9.26)	0.778 ^§^
IOP_OD_ 24-h phase, hour:min	12:24 (11:32; 13:36)	0:12 (23:00; 1:16)	<0.001 ^$^
IOP_OS_ 24-h phase, hour:min	12:40 (11:24; 14:16)	0:48 (23:40; 1:36)	<0.001 ^$^
**Temperature, Sleep and Phase-Alignment Characteristics**			
Bedtime Mean, hour:min	22:08 (21:44; 22:33)	23:02 (21:55; 00:10)	<0.001 ^§^
Mean Time of Awakening, hour:min	5:22 (4:20; 6:15)	5:29 (4:10; 6:47)	0.984 ^§^
Sleep Duration mean, hour:min	7:13 (6:29; 7:58)	6:18 (5:22; 7:14)	<0.001 ^§^
Sleep Phase mean, hour:min	1:45 (1:10; 2:19)	2:15 (1:05; 3:26)	0.002 ^§^
Tb phase, hour:min	14:54 (14:24; 15:32)	19:44 (17:32; 21:12)	<0.001 ^§^
MEQ, Score	60.52 (57.12; 63.92)	66.22 (62.11; 70.32)	0.033 ^§^
IOP_OD_–ASP Ψ, hour:min	9:00 (8:13; 9:48)	4:38 (3:47; 5:32)	<0.001 ^§,^*
IOP_OS_–ASP Ψ, hour:min	9:08 (8:26; 9:51)	3:31 (2:46; 4:16)	<0.001 ^§,^*
IOP_OD_–Tb Ψ, hour:min	−1:08 (−2:09; −0:06)	2:27 (1:02; 3:52)	<0.001 ^§^
IOP_OS_–Tb Ψ, hour:min	−1:21 (−2:18; −0:24)	3:09 (1:30; 4:32)	<0.001 ^§^

IOP_OD_—intraocular pressure, right eye; IOP_OS_—intraocular pressure, left eye; SD—standard deviation; 24-h Amplitude—mean amplitude of best-fitted cosine curve; IOP 24-h phase—IOP phase of best-fitted cosine curve; Tb phase—body temperature phase of best-fitted cosine curve; MEQ Score—Horne–Ostberg’s Morningness–Eveningness Questionnaire score; ASP—average sleep phase; IOP—ASP Ψ-phase lag between IOP and ASP; IOP—Tb Ψ-phase lag between IOP and Tb; Mean values (95% confidence range) are shown. ^§^ Mann–Whitney U test; ^$^ Bingham’s parameter-test; * statistically significant difference between right and left eyes for A-POAG group.

## Data Availability

The data presented in this study are available on request from the corresponding author. The data are not publicly available due to privacy.

## Supplementary Materials

Supplementary materials can be found at https://www.mdpi.com/1422-0067/22/1/359/s1.

## Abbreviations

Ψ	intrinsic phase angle between intraocular pressure and body temperature circadian rhythms
A-POAG	Advanced Primary Open-Angle Glaucoma
ACE	Angiotensin Converting Enzyme
ASP	Average Sleep Phase (of the three-day self-reported sleep assessment)
BMI	Body Mass Index
FLV	Focal Loss Volume
GLV	Global Loss Volume
IOP	Intraocular pressure
MEQ	Morningness-Eveningness Questionnaire
MESOR	Midline Estimating Statistics of Rhythm, or 24-h cosine adjusted mean
OD	right eye (Oculus Dexter)
ONH	Optic Nerve Head
OS	left eye (Oculus Sinister)
NPDS	Non-Penetrating Deep Sclerectomy
PERG	Pattern Electroretinogram
POAG	Primary Open-Angle Glaucoma
RNFL	Retinal Nerve Fiber Layer
RGCs	Retinal Ganglion Cells
SAP mD	Standard Automated Perimetry mean Deviation
SCN	Suprachiasmatic nucleus
S-POAG	Stable Primary Open-Angle Glaucoma
STE	Sinustrabeculectomy
Tb phi	Body temperature circadian phase
