# Supplementary material for: Disruption of 24-Hour Rhythm in Intraocular Pressure Correlates with Retinal Ganglion Cell Loss in Glaucoma
Source: Int J Mol Sci. 2020 Dec 31;22(1):359. doi: 10.3390/ijms22010359 (PMC7795318; doi:10.3390/ijms22010359)
Supplement: Supplementary file 1 [file ijms-22-00359-s001.pdf]

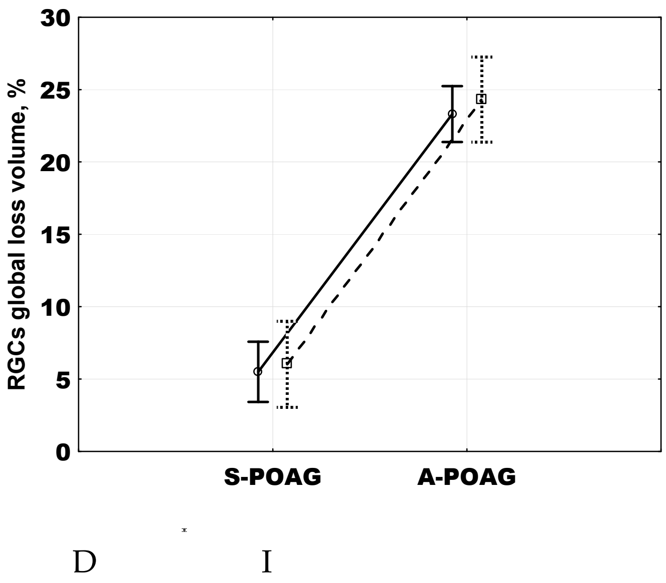

Figure S1. Similar retinal ganglion cells (RGCs, 2-eye mean) global loss volume between *Alu*-repeat deletion carriers, D-carriers (D) and non-carriers (I) of stable or advanced primary open-angle glaucoma (S-POAG and A-POAG) groups; two-eyes mean values are depicted, allele*group interaction ($F_{(1, 15)} = 0.041$, $p = 0.843$). D – *Alu*-repeat deletion carriers (DD and ID genotypes combined); I - non-carriers of *Alu*-repeat deletion.

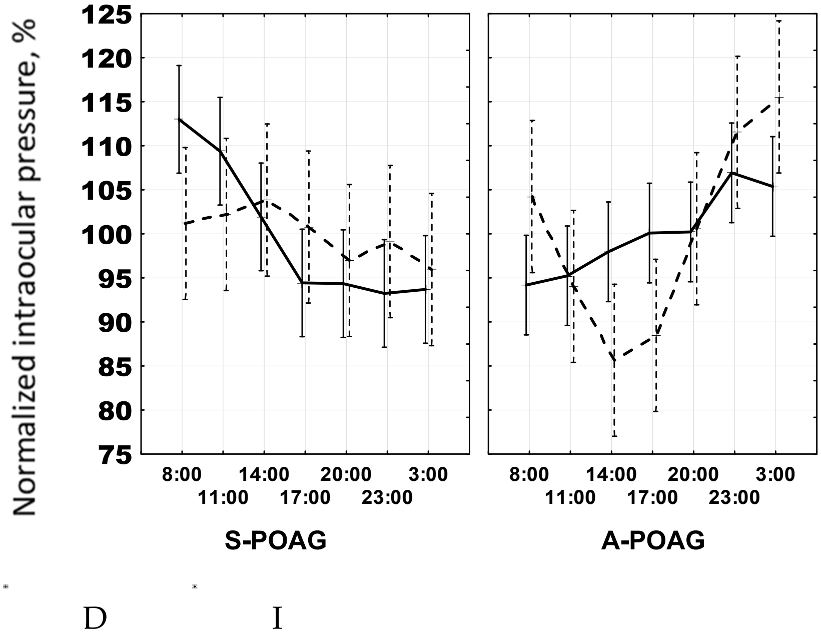

Figure S2. ACE *Alu*-repeat insertion-deletion (I/D) polymorphism defines specific left-eye IOP 24-hour pattern. MANOVA for time*group*allele interaction: $F_{(6,\ 371)} = 3.27$, p=0.004. Normalized data (percentages of the individual IOP averages, IOP%) were used to avoid bias, stemming from different inter-individual / inter-group IOP mean values. D – *Alu*-repeat deletion carriers (DD and ID genotypes combined); I - non-carriers of *Alu*-repeat deletion.

Figure S3. Examples of Optical Coherence Tomography protocols representing patients with stable primary open-angle glaucoma (left) and advanced primary open-angle glaucoma (right).

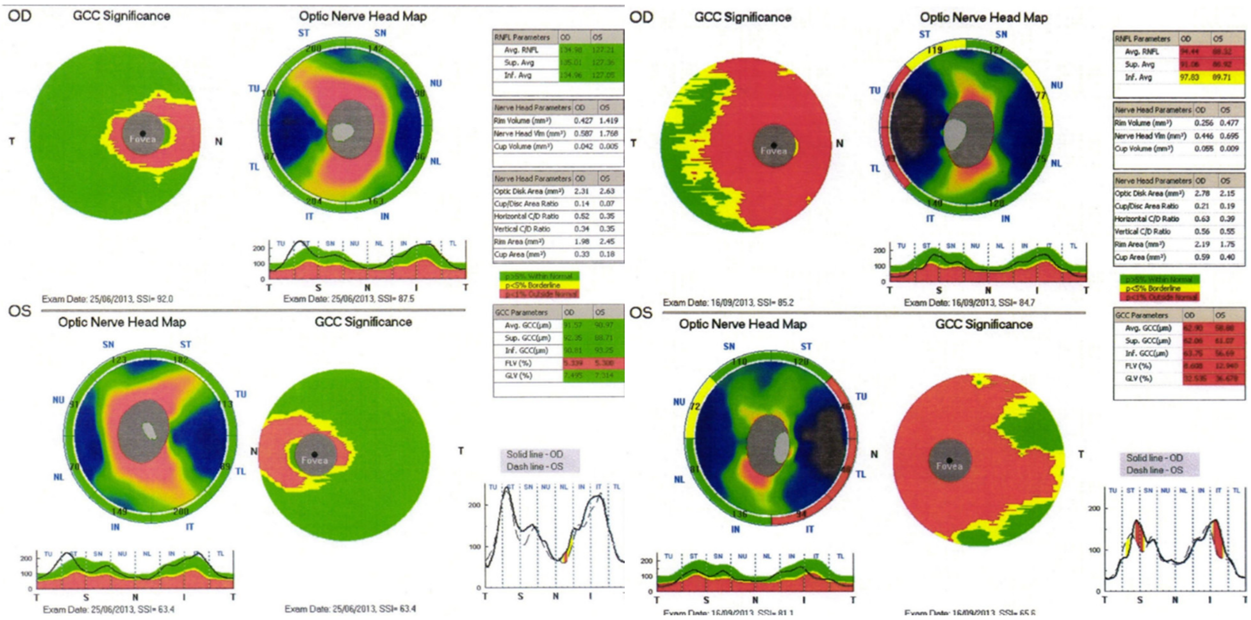

Table S1. Comparative analysis of IOP 24-hour patterns in two groups of POAG patients with different drug treatment or surgery.

	Drug treatment			Surgery
	BB	PGA	CAI	NPDS
IOP OD	F=0.18<br>p=0.981	F=0.19<br>p=0.979	F=0.96<br>p=0.452	F=0.11<br>p=0.996
IOP OS	F=0.58<br>p=0.749	F=0.45<br>p=0.846	F=0.09<br>p=0.997	F=0.47<br>p=0.828

Notes: Treatment consisted of daily drug administration or surgery. Results from MANOVA testing for TIME*GROUP (*S-POAG/A-POAG*)*TREATMENT (*yes/no*) interaction; *IOP – intraocular pressure; POAG – primary open-angle glaucoma: S – stable; A – advanced; BB – beta blockers; PGA – prostaglandin F2α analogues; CAI – carbonic anhydrase inhibitors; NPDS – non-penetrating deep sclerectomy.*

Table S2. Clinical and Sleep Characteristics of Patients with Stable and Advanced Primary Open-Angle Glaucoma, engaged in gene polymorphisms study

	**Stable POAG**	**Advanced POAG**	**P value**
Age, yrs.	65.56 (58.29; 72.82)	67.10 (61.21; 72.99)	0.806§
Sex	n=9, 4 women	n=10, 5 women	0.809#
BMI	27.62 (25.62; 29.62)	26.65 (24.40; 28.90)	0.683§
Mean IOP OD, mmHg	14.51 (12.28; 16.74)	21.14 (18.13; 24.15)	0.004§
Mean IOP OS, mmHg	14.87 (12.42; 17.31)	22.70 (19.87; 25.52)	0.001§
Global Loss Volume mean, %	5.67 (4.30; 7.04)	23.61 (21.71; 25.50)	<0.001§
Focal Loss Volume mean, %	2.79 (0.99; 4.58)	10.88 (9.19; 12.56)	<0.001§
SAP mD mean, dB	-1.93 (-2.28; -1.58)	-9.41 (-14.58; -5.23)	0.012§
PERG P50 A mean, μV	2.04 (1.69; 2.39)	1.11 (0.68; 1.53)	0.005§
Bedtime mean, hour: min	22:28 (22:07; 22:50)	22:48 (22:08; 23:39)	0.369§
Waketime mean, hour: min	6:00 (5:36; 6:24)	5:40 (4:58; 6:22)	0.462§
Mean Sleep Duration, hour: min	7:32 (6:57; 8:07)	6:51 (6:24; 7:19)	0.037§
Mean Sleep Phase, hour: min	2:14 (2:00; 2:29)	2:14 (1:35; 2:53)	0.775§
MEQ, Score	59.22 (45.18; 73.26)	61.00 (51.63; 70.37)	0.934§
Tb phi, hour: min	15:41 (14:06; 17:17)	20:03 (17:39; 22:26)	0.010§

BMI – body mass index; IOP OD –24-hour mean intraocular pressure, right eye; IOP OS –24-hour mean intraocular pressure, left eye; SAP mD - Standard Automated Perimetry Mean Deviation; PERG P50 A – Pattern Electroretinogram main positive peak (P50) Amplitude; MEQ Score – Horne-Ostberg's Morningness-Eveningness Questionnaire score; Tb phi –circadian mean phase of body temperature;

Mean (95% confidence range).

§ Mann-Whitney U test; # Pearson's chi-square test
